# PEAK1, acting as a tumor promoter in colorectal cancer, is regulated by the EGFR/KRas signaling axis and miR-181d

**DOI:** 10.1038/s41419-018-0320-8

**Published:** 2018-02-15

**Authors:** Lanlan Huang, Chuangyu Wen, Xiangling Yang, Qiong Lou, Xiaoyan Wang, Jia Che, Junxiong Chen, Zihuan Yang, Xiaojian Wu, Meijin Huang, Ping Lan, Lei Wang, Aikichi Iwamoto, Jianping Wang, Huanliang Liu

**Affiliations:** 1 Guangdong Institute of Gastroenterology, The Sixth Affiliated Hospital, Sun Yat-sen University, Guangzhou, China; 2 Guangdong Provincial Key Laboratory of Colorectal and Pelvic Floor Diseases, The Sixth Affiliated Hospital, Sun Yat-sen University, Guangzhou, China; 3 Institute of Human Virology and Key Laboratory of Tropical Disease Control (Ministry of Education), Sun Yat-sen University, Guangzhou, China; 4 Department of Clinical Laboratory, The Sixth Affiliated Hospital, Sun Yat-sen University, Guangzhou, China; 5 Advanced Clinical Research Center, Institute of Medical Science, University of Tokyo, Tokyo, Japan

## Abstract

PEAK1 is upregulated in multiple human malignancies and has been associated with tumor invasion and metastasis, but little is known about the role of PEAK1 in colorectal cancer (CRC) progression. We investigated the expression pattern, function and regulatory mechanisms of PEAK1 in CRC. Here, we found that PEAK1 is overexpressed in CRC tissues and that high PEAK1 expression predicts poor survival in colon cancer but not rectal cancer. Functionally, silencing PEAK1 inhibits cell proliferation, migration, and invasion in vitro and inhibits the growth of tumor xenografts in nude mice. Mechanistic studies revealed that PEAK1 is induced by epidermal growth factor receptor (EGFR) signaling and that PEAK1 is required for KRas-induced CRC cell growth and metastasis. Furthermore, we demonstrated that miR-181d directly targets PEAK1. Ectopic expression of miR-181d reduces the expression of PEAK1 and inhibits the growth and metastasis of CRC cells in vitro. Clinically, miR-181d is downregulated in CRC samples, and low miR-181d is correlated with poor patient survival. Our study demonstrates the importance of PEAK1 in CRC progression and suggests a potential mechanism by which increasing PEAK1 expression in CRC might be the result of EGFR/KRas signal activation and consequent miR-181d repression.

## Introduction

Colorectal cancer (CRC) is the most common malignant tumor worldwide^1^. Distant metastasis is a major cause of death in CRC patients^2, 3^. Currently, the standard first-line treatments that have shown promising results for metastatic colorectal cancer (mCRC) are cytotoxic chemotherapy and/or targeted therapies^4^. The most commonly used target therapies for mCRC are the monoclonal antibodies cetuximab and panitumumab, both of which can inactivate the EGFR signaling pathway^5^. Unfortunately, primary and secondary resistance after anti-EGFR antibody therapy has emerged. Recent studies have identified mutations in downstream effectors of the EGFR signaling pathway, such as KRas^6–8^ and other genes^9–11^, as the primary cause of resistance. Therapeutic resistance to EGFR blockade could be overcome through combinatorial therapies targeting EGFR downstream genes^9^. Therefore, defining these genes can help guide treatment and improve clinical care.

Pseudopodium-enriched atypical kinase 1 (PEAK1), a non-receptor atypical tyrosine kinase family member KIAA2002 (sgk269), localizes to the actin cytoskeleton and focal adhesions (FAs) and regulates FA turnover^12, 13^. PEAK1 contains multiple tyrosine phosphorylation sites^12^, and one of these, Y1188, can be phosphorylated by EGF signaling. Such phosphorylation results in PEAK1 binding to endogenous SHC1 and mediating EGFR signal output^14^. Studies show that PEAK1 is overexpressed in multiple human malignancies and has an effect on regulating tumor migration and proliferation^12, 15–17^. In summary, PEAK1 is critical for tumor development and is possibly a new therapeutic target for cancer. However, the role of PEAK1 in CRC progression and its regulatory mechanisms remain unclear. In this study, we focused on the mechanism of PEAK1 over-expression during CRC tumorigenesis.

Here, we show that PEAK1 is significantly upregulated in both colon and rectal cancer, and high PEAK1 expression predicts poor survival in colon cancer. Our study further demonstrates the importance of PEAK1 in CRC progression and indicates the activation of the EGFR/KRas signaling axis and repression of miR-181d as a potential mechanism for increased PEAK1 expression in CRC.

## Results

### PEAK1 overexpression is associated with poor prognosis in CRC patients

**Fig. 1:**
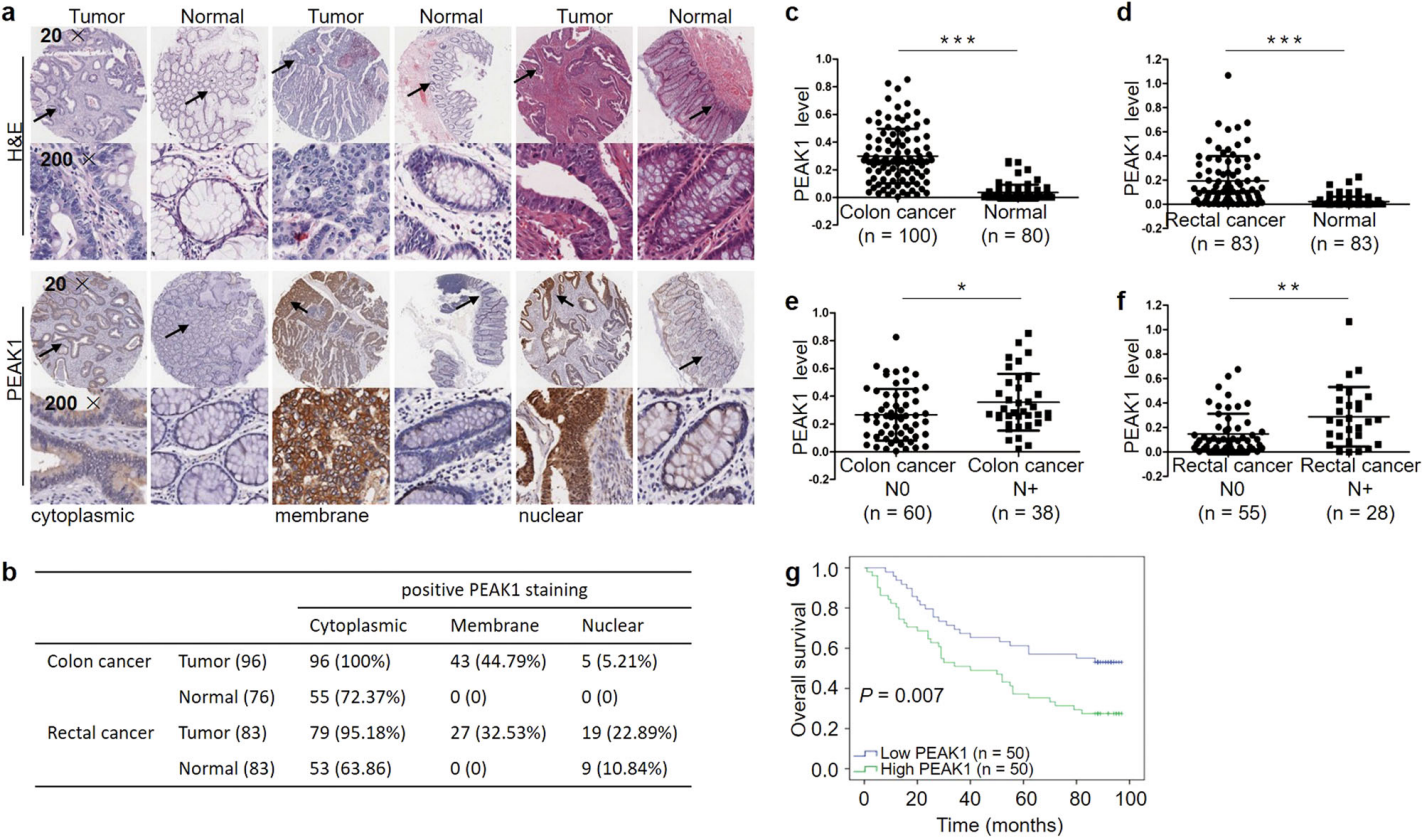
PEAK1 protein expression levels in CRC specimens and prognostic significance. **a** Representative immunohistochemical images of cytoplasmic, membrane and nuclear staining. **b** Summary of PEAK1-positive staining data for colon cancer and rectal cancer. **c** PEAK1 protein expression in 100 colon cancer and 80 normal tissues. Statistical significance was determined by a two-tailed, unpaired Student’s *t*-test. **d** PEAK1 protein expression in 83 pairs of rectal cancer. Statistical significance was determined by a two-tailed, paired Student’s *t*-test. **e**, **f** PEAK1 protein expression in colon cancer tissues without lymph node metastasis (*N* = 60) and with lymph node metastasis (*N* = 38) and rectal cancer tissues without lymph node metastasis (*N* = 55) and with lymph node metastasis (*N* = 28). Statistical significance was determined using a two-tailed, unpaired Student’s *t*-test. **g** Kaplan–Meier analysis of overall survival according to low and high PEAK1 protein expression in 100 colon cancer patients. (*P *<* 0.05, **P < 0.01, ***P < 0.001)

Recent studies have found that PEAK1 is overexpressed in multiple human malignancies and localizes to the actin cytoskeleton and focal adhesions^12, 15^. To better understand the expression and localization of PEAK1 protein in CRC, immunohistochemistry staining was performed in colon and rectal carcinoma tissue microarray (TMA) slides containing tumor tissues and adjacent normal tissues. We found that PEAK1 localized to the cytoplasm, membrane and nucleus (Fig.1a). The positive staining rate was higher in tumor tissues than in normal tissues (Fig.1b), and there was strong immunostaining in tumor tissues (Fig.1a, Supplementary Figure S1a). Significantly higher expression of PEAK1 was observed in tumors compared to adjacent normal tissues (Fig.1c, d). In addition, we found that PEAK1 was markedly upregulated in patients with lymph node metastasis compared to patients without (Fig.1e, f). We then analyzed the correlation of PEAK1 expression with clinicopathological parameters. High PEAK1 expression was significantly associated with advanced clinical stage (Table1). Kaplan–Meier analysis showed that high levels of PEAK1 expression were correlated with poor overall survival in colon cancer (Fig.1g). Further, multivariate Cox regression analysis revealed that PEAK1 expression was an independent prognostic factor for poor survival (Table2). However, PEAK1 expression was not associated with overall survival in rectal cancer (Supplementary Figure S1b, Table S1).

**Table 1:** Correlation between PEAK1 expression and clinical parameters in colorectal cancer patients

Factors	Colon cancer				Rectal cancer			
	*n*	Low PEAK1 (%)	High PEAK1(%)	*P* value	*n*	Low PEAK1 (%)	High PEAK1(%)	*P* value
*Age*				0.149				0.934
<60	20	12 (60%)	8 (40%)		30	15 (50.0%)	15 (50.0%)	
≥60	74	31 (41.9%)	43 (58.1%)		53	27 (50.9%)	26 (49.1%)	
*Gender*				0.753				0.309
Female	44	21 (47.7%)	23 (52.3%)		35	20 (57.1%)	15 (42.9%)	
Male	55	28 (50.9%)	27 (49.1%)		48	22 (45.8%)	26 (54.2%)	
*TNM stage*				0.02^*^				0.001^*^
I+II	60	35 (58.3%)	25 (41.7%)		55	35 (63.6%)	20 (36.4%)	
III+IV	38	13 (34.2%)	25 (65.8%)		28	7 (25.0%)	21 (75.0%)	
*pT*				0.05^*^				0.054
T1+T2	7	6 (85.7%)	1 (14.3%)		22	15 (68.2%)	7 (31.8%)	
T3+T4	89	42 (47.2%)	47 (52.8%)		61	27 (44.3%)	34 (55.7%)	
*pN*				0.02^*^				0.001^*^
N0	60	35 (58.3%)	25 (41.7%)		55	35 (63.6%)	20 (36.4%)	
N1+N2	38	13 (34.2%)	25 (65.8%)		28	7 (25.0%)	21 (75.0%)	
*pM*				0.582				0.309
M0	97	48 (49.5%)	49 (50.5%)		82	42 (51.2%)	40 (48.8%)	
M1	3	1 (33.3%)	2 (66.7%)		1	0 (0.0%)	1 (100%)	

^*^P < 0.05

**Table 2:** Univariate and multivariate analyses of various potential prognostic factors in 100 colon cancer patients

Factors	Univariate analysis		Multivariate analysis	
	HR (95%CI)	*P*	HR (95%CI)	*P*
Age	1.012 (0.988, 1.036)	0.337		
Gender	0.987 (0.590, 1.650)	0.959		
TNM Stage	2.565 (1.625, 4.049)	<0.001^*^	2.365 (1.454, 3.846)	0.001^*^
PEAK1 expression	6.259 (1.718, 22.808)	0.005^*^	5.724 (1.430, 22.912)	0.014^*^

*P < 0.05; *HR*hazard ratio, *CI*confidence interval.

### Downregulation of PEAK1 inhibits CRC cell invasion, migration and proliferation

**Fig. 2:**
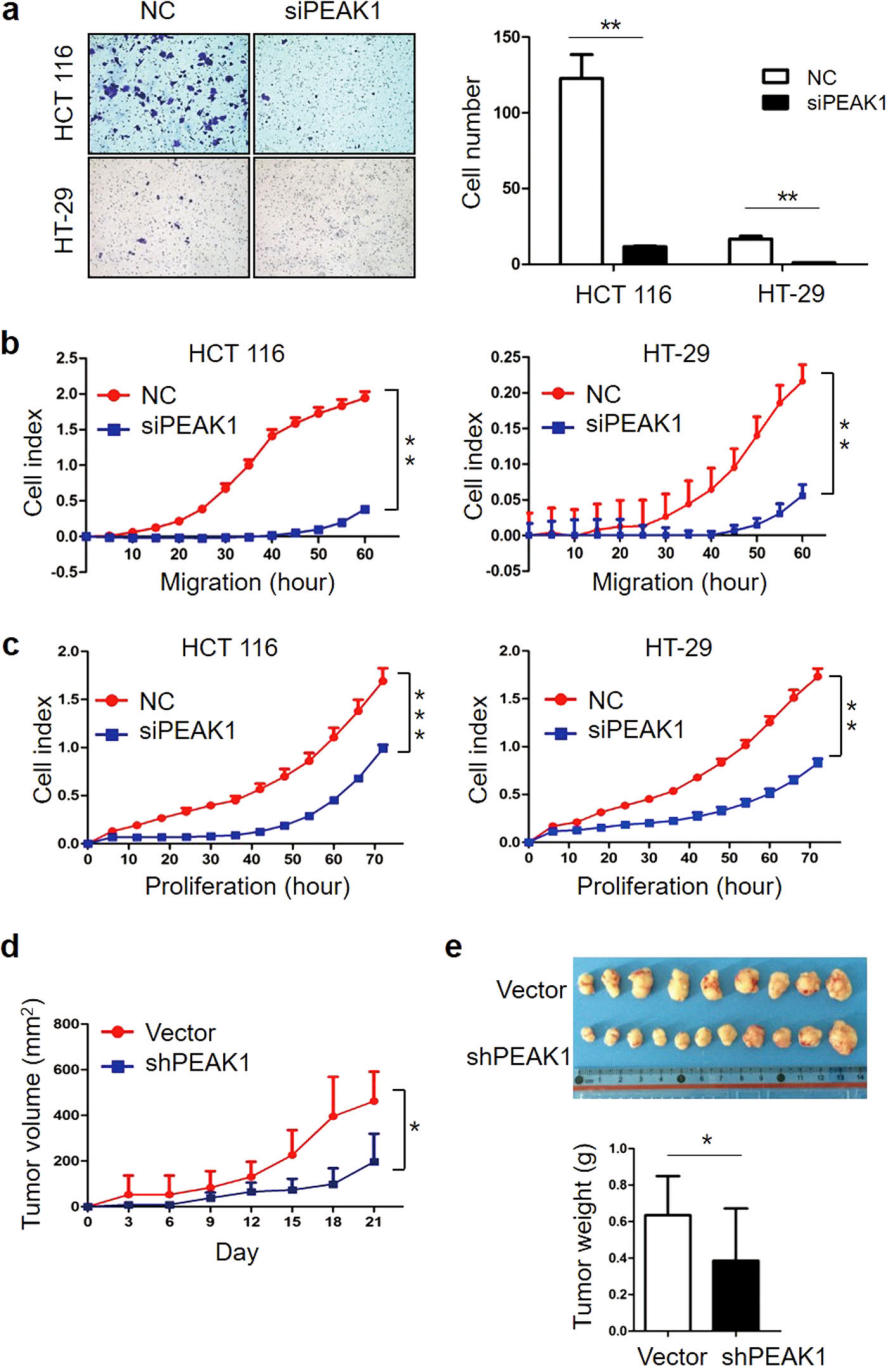
Downregulation of PEAK1 inhibits CRC cell invasion, migration and proliferation. **a** Transwell assays were used to estimate the effects of PEAK1 downregulation on CRC cell invasion abilities. **b**,** c** Real-time migration and real-time proliferation of CRC cells transfected with NC or siPEAK1. The delta cell index indicates electrical impedance measurements. All the above experiments were carried out in triplicate. **d** The tumor growth curve. HCT 116 cells were transfected with pLenti-shRNA or pLenti-vector and subcutaneously injected into nude mice. Statistical significance was determined by Student’s paired *t*-test. (*n* = 12 per group). **e** Photographs of the tumors at day 21 after inoculation with HCT 116 cells transfected with pLenti-shRNA or pLenti-vector (up). Average tumor weight after tumor excision (down). (Data are represented as the mean ± s.d. *P *<* 0.05, **P < 0.01)

PEAK1 was found to be upregulated in CRC and associated with metastasis in breast and pancreatic cancer^12, 15, 16^. To investigate the impact of PEAK1 on the biological properties of CRC cells, HCT 116 and HT-29 cells were depleted of PEAK1 using siRNA and tested for their ability to invade, migrate and grow in vitro. Transfection of HCT 116 and HT-29 cells with siRNA to decrease PEAK1 protein expression (Supplementary Figure S2a) markedly reduced invasion ability compared with NC cells (Fig.2a). Downregulation of PEAK1 also significantly inhibited CRC cell migration and proliferation, as determined by real-time cell migration and proliferation assays (Fig.2b, c). To investigate the role of PEAK1 in tumor growth in vivo, we used pLenti-shPEAK1 to stably knockdown endogenous PEAK1 expression in CRC cell. Transfection of CRC cells with pLenti-shPEAK1 caused decreased PEAK1 protein expression and consequently reduced cell invasion compared with control cells in vitro (Supplementary Figure S2b and c). HCT 116 cells stably transfected with pLenti-shPEAK1 or pLenti-vector were subcutaneously injected into the flanks of nude mice (*n* = 12). HCT 116-shPEAK1**-**formed tumors showed decreased volume and weight compared with pLenti-vector tumors (Fig.2d, e). To further characterize the role of PEAK1 in CRC, we performed a gene-expression microarray assay in HCT 116 cells following siRNA-mediated PEAK1 knockdown. We found that 622 genes were upregulated and 744 were downregulated at least two-fold in HCT 116-siRNA cells (Data set S1). KEGG pathway analysis revealed that the MAPK, Focal adhesions and the PI3K-Akt signaling pathway were the most downregulated pathways in cells transfected with siRNA (Supplementary Figure S3).

### EGFR signaling increases the expression of PEAK1

**Fig. 3:**
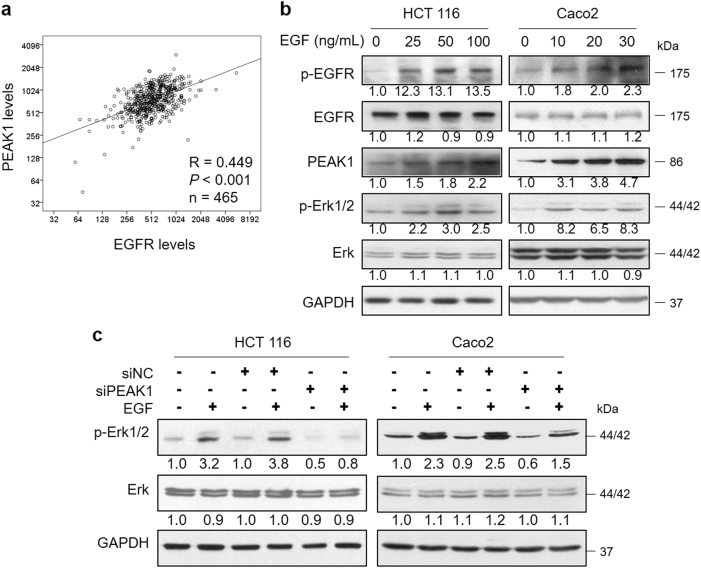
EGFR signaling increases the expression of PEAK1. **a** The correlation between EGFR and PEAK1 expression was evaluated by Spearman’s method. **b** Western blot analyses of EGFR, PEAK1 and Erk in total extracts from CRC cells treated with EGF for 1 h. **c** CRC cells were treated with EGF, followed by siPEAK1 transfection, and then western blot was performed to analyze the Erk levels. These experiments were repeated three times

EGF and other EGF-like ligands trigger EGFR, which activates downstream pro-oncogenic signaling pathways, including the MAPK cascade (RAS-RAF-MEK-ERK) and PI3K-AKT-mTOR pathway, regulating cancer cell survival, growth and motility^18^. As previous studies have suggested, PEAK1 takes part in regulating growth factor receptor signal output^14^. To ascertain this relationship, we first evaluated the expression correlations between EGFR and PEAK1 using public data from The Cancer Genome Atlas (TCGA). Spearman’s correlation analyses showed that EGFR significantly and positively correlated with PEAK1 levels in CRC patients (*n* = 465, Fig.3a). Then, we investigated the effect of EGF stimulation on PEAK1 expression in CRC. HCT 116 and CaCO_2_ were treated with EGF for 1 h and subjected to western blot. The results showed that EGF treatment trigger EGFR/Erk signaling by increased the levels of p-EGFR and p-Erk1/2. PEAK1 expression could be stimulated by EGF in a dose-dependent manner (Fig.3b). CRC cells were treated with EGF followed by siPEAK1 transfection. The cells were harvested after 1 h of EGF treatment. As expected, siRNA-PEAK1 significantly attenuated EGF-induced p-Erk1/2 levels (Fig.3c). Taken together, these results indicate that PEAK1 expression is regulated by EGFR signaling in CRC cells.

### Downregulation of PEAK1 inhibits EGFR/KRas signaling

**Fig. 4:**
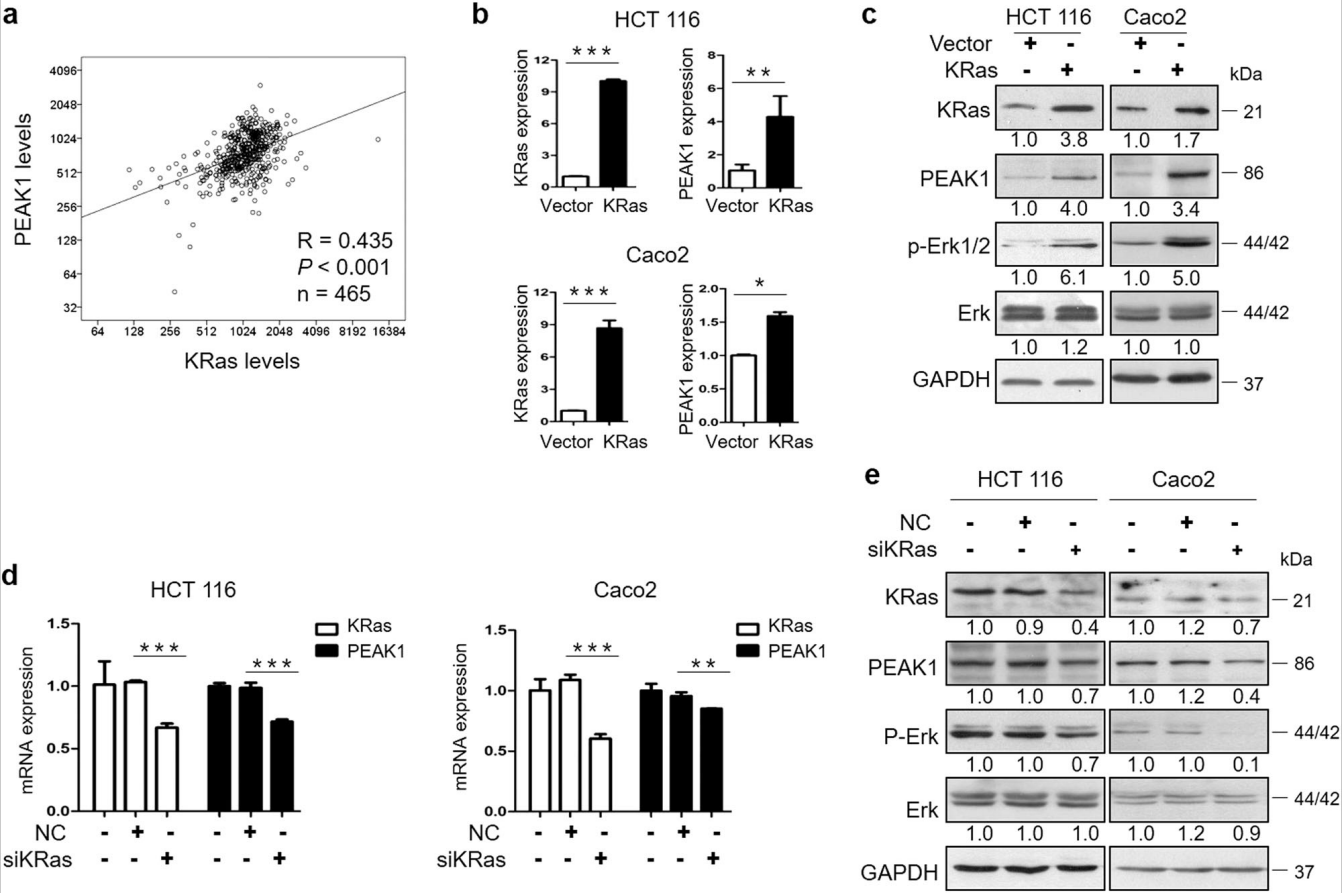
KRas regulates the expression of PEAK1. **a** The correlation between KRas and PEAK1 expression was evaluated by Spearman’s method. **b** qRT-PCR analysis of the expression of KRas and PEAK1 in CRC cells infected with pLenti-KRas or pLenti-vector. **c** Western blot analyses of the expression of KRas, PEAK1 and Erk extracts from CRC cells infected with pLenti-KRas or pLenti-vector. **d** qRT-PCR analysis of the expression of KRas and PEAK1 in CRC cells transfected with siKRas or NC. **e** Western blot analyses of the expression of KRas, PEAK1 and Erk in CRC cells transfected with siKRas or NC. These experiments were repeated three times. (Data are represented as the mean ± s.d. *P *<* 0.05, **P < 0.01, ***P < 0.001)

Over-activation of the EGFR cascade, by KRas gene mutation for example, promotes cell growth, proliferation, migration, and inhibition of apoptosis^10, 19–22^. Our studies have demonstrated that silencing PEAK1 decreased the activity of the EGFR/Erk signaling pathway (Fig.3c, Supplementary Figure S3). Hence, to explore whether KRas regulates PEAK1 expression in CRC, we evaluated the correlation of the expression of KRas and PEAK1. Data from TCGA showed that PEAK1 significantly and positively correlated with KRas levels in CRC patients (*n* = 465, Fig.4a). To test the effects of KRas on endogenous levels of PEAK1 in CRC cells, we used qRT-PCR and western blot assays to measure the mRNA and protein levels of PEAK1 in HCT 116 and CaCO_2_ cells infected with a pLenti- KRas or siRNA-KRas. As shown in Fig.4b,c, overexpression of KRas by pLenti-KRas significantly increased PEAK1 mRNA and protein expression and the levels of p-Erk1/2. Downregulation of KRas by siRNA significantly decreased PEAK1 mRNA and protein levels and the levels of p-Erk1/2 (Fig.4d, e). Taken together, these results demonstrate that KRas induces PEAK1 expression in CRC.

### Downregulation of PEAK1 impairs KRas-induced growth and metastasis in vitro

**Fig. 5:**
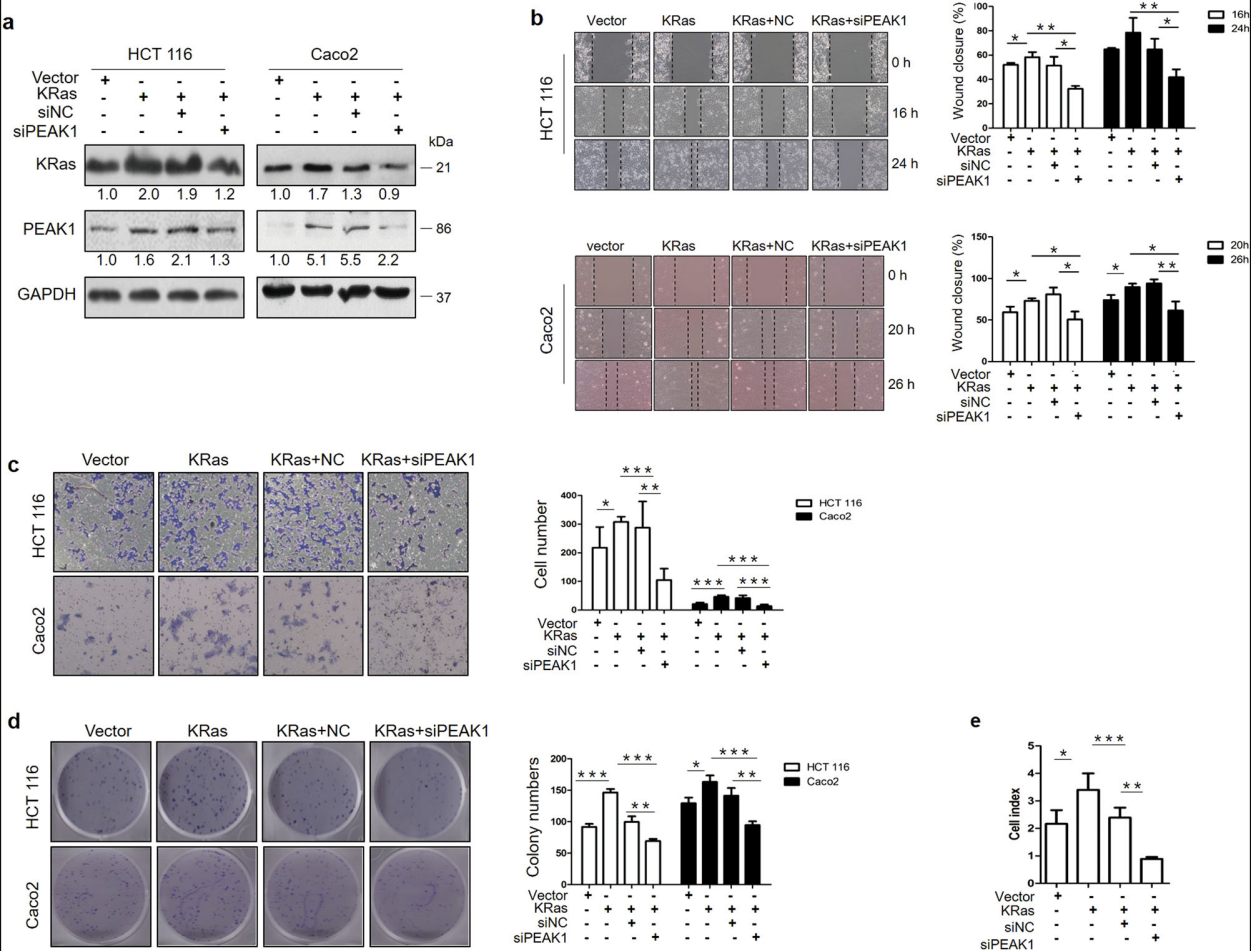
Downregulation of PEAK1 impairs KRas-induced growth and metastasis in vitro. **a** KRas-overexpressing HCT 116 cells were transfected with siPEAK1 for 72 h, and western blot analyses of KRas and PEAK1 protein expression levels were performed. **b** Wound-healing assays were performed to investigate the effects of siPEAK1 on the migration ability of KRas-overexpressing cells. **c** Transwell assays were performed to estimate the effects of siPEAK1 on the invasion abilities of KRas-overexpressing cells. **d**, **e** Cell colony formation and real-time proliferation assays showed the effects of siPEAK1 on cell growth in KRas-overexpressing cells. These experiments were performed in triplicate. (Data are represented as the mean ± s.d. *P *<* 0.05, **P < 0.01, ***P < 0.001)

The above results strongly suggested that PEAK1 expression might be necessary for the KRas-induced biological properties of CRC cells. Therefore, we investigated whether knocking down PEAK1 expression could impair motility and growth in KRas-overexpressing cells. Western blot showed that transfection with siRNA-PEAK1 significantly attenuated KRas-upregulated PEAK1 levels (Fig.5a). Then, we tested the effect of PEAK1 knockdown on cell motility by Wound-healing assay and Matrigel invasion assay. As shown in Fig.5b, c, transfection with pLenti-KRas increased cell migration and invasion, whereas knocking down PEAK1 significantly reduced KRas-induced cell migration and invasion. Next, we tested the effect of PEAK1 knockdown on KRas-induced growth. As shown in Fig.5d, e, KRas promoted colony formation and proliferation. In contrast, downregulation of PEAK1 attenuated KRas-induced growth (Fig.5d, e). Taken together, these findings show that PEAK1 is necessary for KRas-induced invasion, migration, colony formation and proliferation in CRC cells.

### PEAK1 is a direct target of miR-181d in CRC cells

**Fig. 6:**
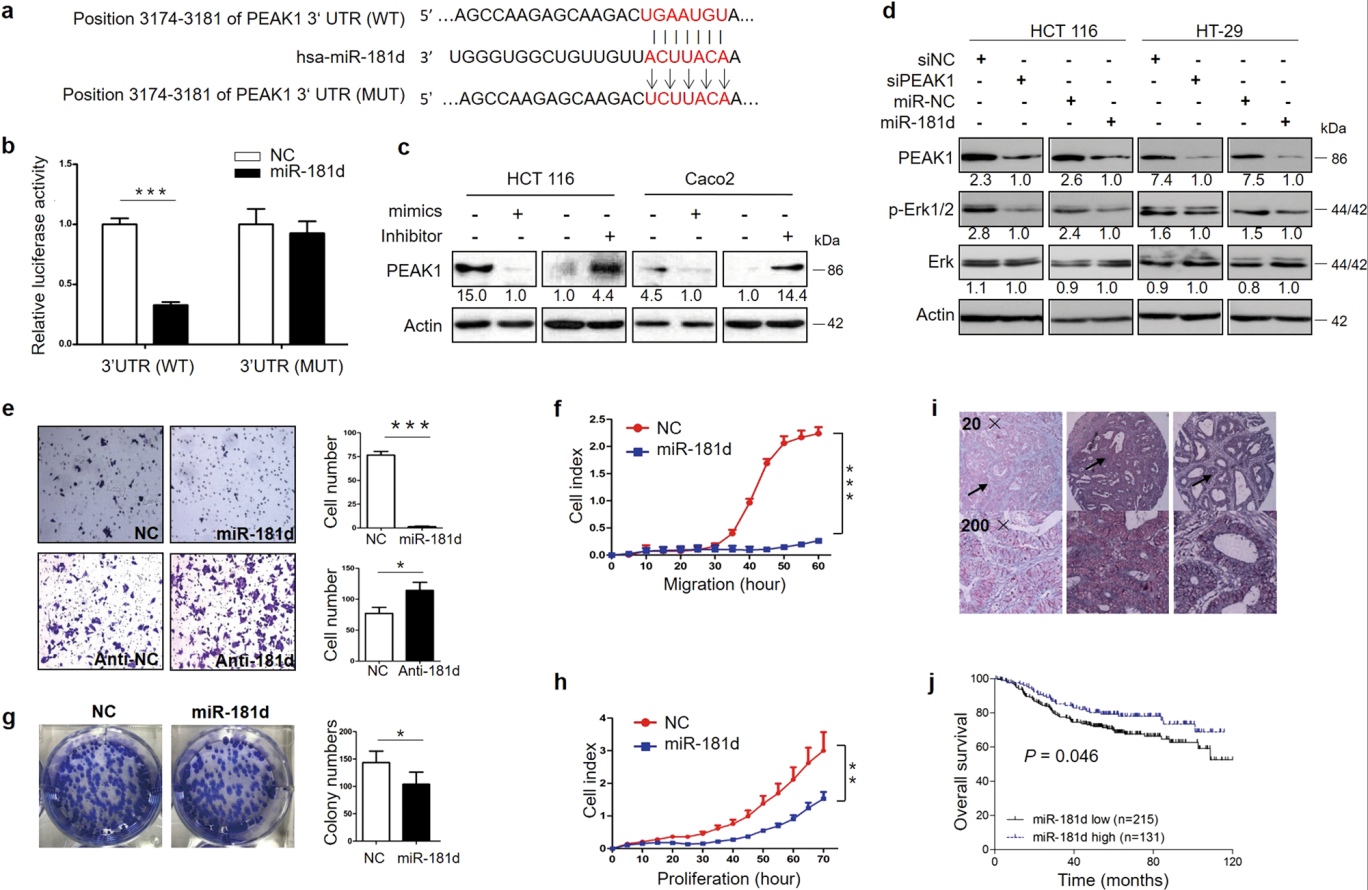
miR-181d targets PEAK1 and as a tumor suppressor in CRC. **a** The conserved miR-181d binding sequence of PEAK1 or its mutated form was inserted into the pMIR reporter. **b** Dual luciferase reporter assay. Luciferase reporter constructs containing wild-type or mutated PEAK1 3′ UTRs were co-transfected with miR-181d mimics or NC into HCT 116 cells. Relative firefly luciferase expression was normalized to Renilla luciferase. **c** Western blot to measure PEAK1 protein levels in CRC cells transfected with miR-181d mimics or inhibitor for 72 h. **d** Western blot showed that overexpression of miR-181d or knockdown of PEAK1 downregulated p-Erk1/2 levels. These experiments were repeated three times. **e** The invasion ability of HCT 116 cells infected with pLenti-miR-181d or anti-miR-181d was analyzed by Transwell assays. **f** Real-time migration assays showed the effects of miR-181d on cell migration. **g**, **h** Cell colony formation and real-time proliferation assays showed the effects of miR-181d on cell growth. These experiments were repeated three times. **i** Representative images for in situ hybridization analysis of miR-181d. No in situ hybridization signal was obtained in the absence of the DIG-labeled probe. Positive staining was expressed as blue–violet. (**j**) Kaplan–Meier analysis of overall survival according to low and high miR-181d expression in 353 CRC patients. (Data are presented as the mean ± s.d. *P *<* 0.05, **P < 0.01, ***P < 0.001)

Linear regression analysis revealed that EGFR (*r*^*2*^ = 0.163) and KRas (*r*^*2*^ = 0.063) do not completely explain the expression pattern of PEAK1 in clinical CRC specimens. Here, we investigated whether miRNAs regulate levels of PEAK1 in CRC cells. To find miRNAs associated with the regulation of PEAK1 expression, a bioinformatics search was performed for potential miRNAs targeting PEAK1 mRNA using common databases such as miRWalk and TargetScan. The results showed that miR-181d was a potential miRNA targeting PEAK1 because miR-181d incompletely complemented the 3′UTR region of PEAK1 (Fig.6a). To verify whether PEAK1 was a direct target of miR-181d, we cloned the 3′UTR region of PEAK1 mRNA, which included the predicted miR-181d recognition site, and then inserted it into a luciferase reporter plasmid. The miR-181d binding site in the 3′UTR region of PEAK1 was mutated to obtain the 3′UTR-MutPEAK1-luc plasmid (Fig.6a). Transient transfection of wild-type PEAK1-luc reporter with miR-181d mimics into HCT 116 cells led to a significant decrease in luciferase activity compared to the activity of NC (Fig.6b). However, the decrease in luciferase activity of Mutant MutPEAK1-luc compared to that in the NC group was not significant (Fig.6b). To determine whether miR-181d affects PEAK1 expression in the intracellular environment in CRC, the expression of PEAK1 was evaluated in HCT 116 and CaCO_2_ cells following transfection with either miR-181d mimics or inhibitors (anti-miR-181d). The transfection efficiency was confirmed by qRT-PCR (Supplementary Figure S4a). Transfection with miR-181d mimics resulted in a significant reduction of PEAK1 protein expression (Fig.6c). Downregulation of miR-181d using anti-miR-181d was associated with significantly higher expression of PEAK1 protein (Fig.6c). We also investigated the correlation between the expression levels of miR-181d and the EGFR/Erk signaling pathway. As expected, miR-181d over-expression led to a significant decrease in PEAK1 protein levels and in the levels of p-Erk1/2 (Fig.6d). The results make it evident that miR-181d affects PEAK1 expression by directly binding to the 3′UTR region of PEAK1 and validating that PEAK1 is a direct target of miR-181d.

### Ectopic expression of miR-181d decreases the invasive, migratory and proliferative capacities of CRC cells in vitro

Our studies demonstrated that miR-181d targets PEAK1. Therefore, we next investigated the role of miR-181d in CRC cells. HCT 116 cells were stably infected with the pLenti-miR-181d or pLenti-vector (Supplementary Figure S4b). Overexpressing miR-181d resulted in a reduction in PEAK1 expression (Supplementary Figure S4c). Invasion assays showed that ectopic miR-181d expression significantly decreased the invasive ability of HCT 116 cells (Fig.6e). However, transfection of CRC cells with the miR-181d inhibitor enhanced cell invasion compared with NC cells (Fig.6e). Real-time cell migration assays showed a reduced migration of miR-181d-overexpressing cells (Fig.6f). Colony formation and real-time cell proliferation assays showed that ectopic miR-181d expression decreased colony formation and cell proliferation (Fig.6g, h). Taken together, these findings indicate that miR-181d acts as a tumor suppressor in CRC.

### miR-181d is an independent prognostic factor for CRC

Our studies have shown that high PEAK1 expression is associated with poor overall survival in colon cancer. To further investigate the clinical pathology and prognostic significance of miR-181d expression, in situ hybridization (ISH) staining for miR-181d was performed on 353 CRC samples in a tissue microarray. The positive staining of tissue was expressed as blue–violet (Fig.6i). As shown in Supplementary Table S2, miR-181d expression was significantly correlated with tumor location, local relapse and TNM stage. The Kaplan–Meier curve and log-rank test showed that downregulation of miR-181d was significantly associated with poorer overall survival in CRC (Fig.6j). In multivariate analysis using the Cox proportional hazards model, miR-181d expression was found to be an independent prognostic factor for CRC (Supplementary Table S3).

## Discussion

In this study, we found that PEAK1 was a prognosis-associated marker that was upregulated in CRC. Our immunohistochemistry results showed that PEAK1 localized to the cytoplasm, membrane and nucleus. These findings are consistent with the results of the work of Wang et al.^12^, who reported that PEAK1 localized to the actin cytoskeleton and FAs in migrating cells. We also found that PEAK1 was exclusively localized to the membrane of CRC cells, indicating that spatiotemporal regulation of PEAK1 was disrupted in tumor tissues. In addition, we showed that PEAK1 protein expression was increased in CRC tissues in comparison with normal tissues and correlated with TNM stage. Subsequent analyses involving 264 CRC patients from the TCGA llluminaGA_RNASeqV2 data set confirmed that high PEAK1 expression was associated with poor overall survival in CRC (Supplementary Figure S1c, Table S4 and 5). In two cohort studies of CRC, high levels of PEAK1 expression were associated with poorer overall survival, indicating that PEAK1 has a critical role in CRC development.

Previous studies have identified PEAK1 as a positive regulator of cell growth and metastasis in breast cancer and pancreatic cancer^15, 17, 23^. However, the role of PEAK1 in CRC remains unknown. Here, we found that downregulation of PEAK1 inhibited CRC cell invasion, migration, and proliferation. We then investigated possible pathways by which PEAK1 could be involved in CRC. Our studies showed that down-regulating PEAK1 inactivated EGFR signaling (MAPK and PI3K-Akt signaling pathway) and the focal adhesion signaling pathway (Supplementary Figure S3). Recently, numbers of studies have shown roles for activated MAPK and PI3K-Akt signaling in the regulation of metastasis and proliferation in cancer^24–29^. The above studies suggest that downregulation of PEAK1 expression decreases EGFR signaling, thereby decreasing CRC cell invasion, migration, and proliferation.

Previous studies have shown that PEAK1 takes part in the EGFR signal output^14^. PEAK1 protein expression is positively regulated by KRas/Src and KRas/eIF5A signaling in pancreatic cancer^15, 30, 31^. Our gene-expression microarray assay results showed that downregulation of PEAK1 inactivated a number of pathways, including the MAPK and PI3K-Akt signaling pathways, indicating that PEAK1 may be involved in EGFR signaling transduction. Our studies also showed that PEAK1 expression was significantly positively correlated with EGFR and KRas levels in CRC patients. EGF stimulation showed that EGFR induced PEAK1 expression, while inhibiting the expression of PEAK1 impaired EGF/Erk signaling stimulated by EGF. Furthermore, we found that KRas could induce PEAK1 expression and that PEAK1 was necessary for KRas-induced growth and metastasis in CRC. Together, these results demonstrate that PEAK1 is under the regulation of the EGFR/KRas signaling axis and promotes an aggressive phenotype in CRC.

Multiple linear regression analysis revealed that EGFR and KRas did not completely explain the expression pattern of PEAK1 in clinical CRC specimens. In fact, our statistical analysis using the TCGA data set showed elevated PEAK1 mRNA expression in normal tissue compared with cancer tissue (Supplementary Figure S1d). Possible explanations for these apparently opposite results include differences in mRNA and protein levels of PEAK1. For example, in breast cancer, the PEAK1 expression pattern was not reflected in the relative mRNA levels, indicating that elevated PEAK1 expression in breast cancer cells must be mediated via a post-transcriptional or post-translational mechanism^16^. MicroRNAs (miRNAs) are small (19–25 nt), noncoding, regulatory RNAs that regulate gene expression by complementary base pairing with the 3′ untranslated region (UTR) of target messenger RNAs (mRNAs), causing mRNA degradation or suppressing mRNA translation^32, 33^. Up to 30% of human genes appear to be conserved miRNA targets^33^. miRNAs were reported to be associated with pathogenesis and could be used as diagnostic and prognostic biomarkers in many human cancers^34–38^. miRNAs are known to regulate gene expression at the post-transcriptional level^32, 39^. Our study investigated the potential involvement of a miRNA-mediated mechanism in the increased expression of PEAK1 in CRC. We performed a bioinformatics search for potential miRNAs targeting PEAK1 mRNA and found that miR-181d had the highest predictive scores, indicating that miR-181d might directly target PEAK1. The Luciferase activity assay performed later confirmed our suspicion. Together, these findings suggest that low expression of miR-181d leads to high expression of PEAK1 in CRC.

It has been reported that miR-181d is downregulated in glioma and acts as a tumor suppressor by targeting KRas and Bcl-2^40^. High expression of miR-181d was associated with improved overall survival in glioblastoma^41, 42^. Exogenous over-expression of miR-181d inhibited the proliferation of pancreatic cancer cells^43^. Guo et al.^44^ reported that miR-181d functions as a tumor promoter in CRC. To better understand the role of miR-181d in CRC, the clinical significance and biological function of miR-181d were analyzed. Our results showed that miR-181d expression was significantly correlated with local relapse and TNM stage, and the downregulation of miR-181d was significantly associated with poorer overall survival in CRC. In addition, gain-of-function and loss-of-function assays were performed to assess the effect of miR-181d on CRC invasion and metastasis. The results showed that silencing miR-181d upregulated PEAK1 and strengthened cell invasion in vitro, whereas overexpressing miR-181d inhibited PEAK1 expression as well as cell proliferation, invasion and migration in vitro. Hence, miR-181d is an important tumor suppressor miRNA in CRC invasion and metastasis, and PEAK1 is downstream effector of miR-181d in its target network. To conclude, we show a novel regulatory mechanism of PEAK1 expression in CRC in which miR-181d suppresses its direct target, PEAK1, in turn regulating CRC invasion and metastasis.

Together, our data demonstrate an association between PEAK1 expression and worse prognosis in CRC and the mechanism of PEAK1 over-expression during CRC tumorigenesis.

## Materials and methods

### Human samples

CRC TMA slides used for immunohistochemistry analysis of PEAK1 protein expression were purchased from Shanghai Outdo Biotech (Shanghai, China). CRC TMA slides used for ISH analysis of miR-181d expression were obtained from the tumor bank of the Department of Pathology of the First Affiliated Hospital, Sun Yat-sen University (Guangzhou, China). The procedure for human sample collection was approved by the Ethical Committee of Sun Yat-sen University (Guangzhou, China), and written informed consent was obtained from all of the patients. All data were analyzed anonymously, and all experiments were in compliance with the Helsinki Declaration.

### Immunohistochemistry of PEAK1

Immunohistochemistry was performed using a PEAK1 antibody (Sigma-Aldrich, St. Louis, MO, USA). The detailed procedures are described in the Supplemental materials and methods.

### Western blot analysis

Cell lysates (40 μg protein/line) were separated on a 10% SDS-PAGE gel and transferred to a polyvinylidene fluoride (PVDF) membrane (Millipore, Billerica, MA, USA). The blotted membranes were blocked with 5% skim milk or 5% bovine serum albumin and incubated overnight at 4 °C. Anti-PEAK1 (86 kDa, Abnova, Taipei, Taiwan), anti-EGFR (Tyr1173, 175 kDa), anti-EGFR (175 kDa), anti-KRas (21 kDa), anti-phospho-p44/42 Erk (Thr202/Tyr204, 44/42 kDa) anti-p44/42 Erk (44/42 kDa) (Cell Signaling Technology, Danvers, MA, USA), anti-β-actin (43 kDa) and anti-GAPDH (36 kDa) (Abcam, Cambridge, UK) antibodies were used. The detailed procedures are described in the Supplemental materials and methods.

### In situ hybridization of miR-181d

ISH was performed as described previously^45^. Briefly, ISH was performed using a hsa-miR-181d probe from Exiqon (miRCURY LNA Detection probe, 250 pmol, 5′-DIG and 3′-DIG labeled). Detection of the probe was carried out using anti-digoxigenin-AP (Roche, Germany), and the hybridized probes were detected by applying a BCIP/NBT Alkaline Phosphatase Color Development Kit. No-probe controls were included for each hybridization procedure. Images were taken using a Leica DMI 4000B inverted microscope (Leica Micro-systems, Wetzlar, Germany). ISH staining of the image was analyzed using Image-Pro-Plus 6.0 (Media Cybernetics, CA, USA).

### Cell proliferation, migration and invasion assays

Cell proliferation and migration assays were performed on the xCELLigence system from ACEA Biosciences. Cell invasion assays were performed on Transwell chambers pre-coated with Matrigel (BD Bioscience, San Jose, CA, USA). Detailed procedures are described in the Supplemental Materials and Methods.

### Animal study

All animal studies were conducted in accordance with German animal welfare law and approved by the Institutional Animal Care and Use Committee of Sun Yat-sen University. HCT 116 cells transfected with pLenti-shRNA or pLenti-vector were harvested by trypsin, washed with PBS, and resuspended in RPMI medium supplemented with 10% FBS. A total of 5 × 10^6^ cells were subcutaneously transplanted into the flanks of 5-week-old nude mice (six mice per group). Injections were performed in both flanks of each animal. Tumor volumes were measured with calipers and calculated as length × width^2^ × 0.4. The tumor sizes were measured at 3-day intervals as soon as the tumors were measurable. On day 21, the tumor masses were measured, excised, and further analyzed.

### Statistical analysis

Statistical analysis was performed using SPSS18.0 software (SPSS, IBM, Chicago, IL, USA). Data are expressed as the mean ± s.d., and statistical significance was determined with Student’s *t*-tests. Statistical comparisons between groups were analyzed using Student’s paired *t*-test. *P-*values <0.05 were considered statistically significant. Correlations between clinicopathological features and PEAK1/miR-181d expression were calculated according to the Chi-square test. The cumulative survival time was calculated utilizing the Kaplan–Meier method and analyzed with the log-rank test. Univariate and multivariate analyses were performed based on the Cox proportional hazards regression model.

## Electronic supplementary material

Supporting information

Data set 1
