# Supplementary material for: PEAK1, acting as a tumor promoter in colorectal cancer, is regulated by the EGFR/KRas signaling axis and miR-181d
Source: Cell Death Dis. 2018 Feb 15;9(3):271. doi: 10.1038/s41419-018-0320-8 (PMC5833579; doi:10.1038/s41419-018-0320-8)
Supplement: Supplementary file 1 — Supporting information [file 41419_2018_320_MOESM1_ESM.docx]

**Supporting Information**

**PEAK1, acting as a tumor promoter in colorectal cancer, is regulated by the EGF/KRas signaling axis and miR-181d**

Lanlan Huang^1,2,a^, Chuangyu Wen^1,2,a^, Xiangling Yang^1,2,a^, Qiong Lou^1,2,3,4^, Xiaoyan Wang^1,2^, Jia Che^1,2,3,4^, Junxiong Chen^1,2^, Zihuan Yang^1,2^, Xiaojian Wu^1,2^, Meijin Huang^1,2^, Ping Lan^1,2^, Lei Wang^1,2^, Aikichi Iwamoto^5^, Jianping Wang^1,2*^, Huanliang Liu^1,2,3,4*^

# Supplementary Material and Methods

**Tissue Specimens and Patient Information.** CRC tissue microarray (TMA) slides used for immunohistochemistry (IHC) analysis of PEAK1 protein expression was purchased from Shanghai Outdo Biotech (Shanghai, China). The colon cancer TMA slide (HColA180Su09) contains 100 tumor tissues and 80 adjacent normal tissues. The rectal cancer TMA slide (HRec-Ade180Sur-04) contains 89 tumor tissues and 89 adjacent normal tissues. These patients who had been diagnosed with CRC and underwent initial surgical resection for CRC between January 2006 and November 2008 were follow-up by telephone or letters from surgery up until September 2014 to collect general information, pathology reports, and information regarding the patients’ condition after surgery. CRC TMA slide used for *In situ* hybridization (ISH) analysis of miR-181d expression were obtained from the tumor bank of the Department of Pathology of the First Affiliated Hospital, Sun Yat-sen University (Guangzhou, China). These 353 patients who had been diagnosed with CRC and underwent initial surgical resection for CRC between January 2000 and November 2006 were follow-up by telephone or letters from surgery up until April 2010 to collect general information, pathology reports, and information regarding the patients’ condition after surgery. The procedure of human sample collection was approved by the Ethical Committee of Sun Yat-sen University (Guangzhou, China), and written informed consent was obtained from all of the patients. All data were analyzed anonymously and all experiments were in compliance with the Helsinki Declaration.

**TCGA Dataset.** The TCGA RNASeq and miRNASeq dataset with clinical information was downloaded on 04/20/2016.

**Cell Culture and Treatment.** Human CRC cell lines were purchased from Culture Collection of Chinese Academy of Science (Shanghai, China). Cells were cultured in RPMI 1640 or DMEM medium supplemented with 10% fetal bovine serum (vol/vol) and 1% penicillin-streptomycin (Gibco, Grand Island, NY, USA) at 37°C in 5% CO2. miRNA mimics and antisense oligonucleotides (inhibitor) were used to overexpression or knockdown of miR-181d levels. miR-181d mimics, inhibitor, siRNA and negative control were obtained from RiboBio. CRC cells were transfected with 50nM of miRNA/siRNA using Lipofectamine 3000 (Invitrogen, Carlsbad, CA, USA). For EGF (PEPROTECH, USA) treatment, cells were serum starved for 12 h and then treated with EGF for 0.5 to 46 h.

**Quantitative Real-Time PCR.** Total RNA from cultured cells was extracted using Trizol Reagent (Invitrogen, Carlsbad, CA, USA). Total RNA was reverse-transcribed to cDNA using the PrimeScript^TM^RT reagent Kit and random primers (Takara, Dalian, China). qRT-PCR for KRas and PEAK1 mRNA was analyzed by SYBR Premix Ex Taq^TM^ II (Takara), and normalized to GAPDH mRNA, according to the manufacturer's instructions. Expression of mature miR-181d was analyzed using All-in-One miRNA qRT-PCR Detection Kit (GeneCopoeia, USA) according to the manufacturer’s instructions. Small endogenous nucleolar RNU6B was used as control for miRNA normalization. All the experiments were done in triplicate. Real-time PCR was performed using an Applied Biosystems 7900 Real-time PCR system (Applied Biosystems, Foster City, CA, USA). Gene expression △Ct values from each sample were calculated by normalizing with an internal control (RNU6B/GAPDH), and relative expressions were calculated using the formula 2^-△△Ct^ values. Differences between gene expression levels among two groups were evaluated using a t-test; *P* < 0.05 was considered signiﬁcant. Statistics were performed with SPSS.

**Western Blot Analysis.** Cells were collected from cultured dishes and were lysed in a RIPA lysis buffer (Cell Signaling Technology, Danvers, MA, USA) supplemented with inhibitors of proteases..The procedures for western blot were performed as described previously ^1^. Briefly, equal amounts of protein were separated by 10% sodium dodecyl sulphate-polyacrylamide gel electrophoresis (SDS-PAGE) and transferred to a polyvinylidene fluoride (PVDF) membrane (Millipore, Billerica, MA, USA). The membranes were then blocked with 5% skim-milk or 5% bovine serum albumin for 1 hour at room temperature, and incubated with primary antibodies overnight at 4°C. The next day, after incubating with horseradish peroxidase-conjugated secondary antibodies for 1 hour at room temperature, the signals were detected using a chemiluminescence detection kit ECL (Santa Cruz biotechnology). anti-PEAK1 (86 kDa, 1:500 dilution, Abnova, Taipei, Taiwan), anti-EGFR (Tyr1173, 175kDa), anti-EGFR (175kDa), anti-KRas (21 kDa), anti-phospho-p44/42 Erk (Thr202/Tyr204, 44/42 kDa) and anti-p44/42 Erk (44/42 kDa) (1:1000 dilution, Cell Signaling Technology), β-actin (43 kDa) and GAPDH (36 kDa) (1:1000 dilution, Abcam, Cambridge, UK) antibodies were used.

**Immunohistochemistry of PEAK1.** Commercially available tissue microarray (TMA) slides (HColA180Su09, HRec-Ade180Sur-04, Shanghai Outdo Co., Shanghai, China) was applied to evaluate the expression of PEAK1. Specific primary antibodies against PEAK1 (Sigma-Aldrich, USA) was used for IHC analysis. IHC staining was performed as described previously ^2^. Briefly, TMA slides were incubated in dry oven at 63 °C for about 1 hour. After de-paraffinization and rehydration, antigen retrieval was performed by boiling sections in 0.1mol/L citrate acid solution (pH = 6.0). Endogenous peroxidase activity was blocked using 0.3% H_2_O_2_ for 15 min at room temperature. The TMA slides were blocked with 3% BSA in PBS for 30 min and subsequently incubated with PEAK1 antibody (1:2000) overnight at 4°C. Antibody staining was visualized with diaminobenzidine (DAKO) and hematoxylin (Sigma) counterstain. Immunohistochemical staining of the image was analyzed by using the Image Pro-Plus (version 6.0, Media Cybernetics, Silver Spring, USA) introduced by Xavier ^3^. In brief, the tumor area was selected as the area of interest (AOI), and the area sum and integrated optical density (IOD) of the AOI were selected as the measurement parameters. PEAK1 expression index equalled the quotient between the IOD and the total area of AOI. Finally, the mean expression index for each duplicate was used for statistical analysis. In addition, staining rate was classified separately for the nuclear, membrane and the cytoplasm. For each tissue specimen, three areas of different staining intensity are selected. In each area, 100 cells were analyzed and the average percentage of positively stained cells of the three areas would be the final staining rate. The expression of PEAK1 was graded as positive when 10% of tumor cells showed immunopositivity.

**Invasion Assay.** Transwell chambers precoated with Matrigel (BD Bioscience, San Jose, CA, USA) were used to perform the invasion assay. Cells were cultured in serum-free medium in the upper chambers of a Transwell plate, which are separated from the lower chambers with permeable 8.0 mm polycarbonate membranes; medium containing 10% FBS served as the attractant in the lower chambers. After 36 hours, the cells were fixed with 4% Polyoxymethylene and stained with crystal violet. Non-migrating cells on the upper side of the membrane were gently wiped off, and the stained cells on the lower side were observed under a microscope. The number of migrating cells in five fields per chamber was counted and average values were calculated.

**Real-Time Cell Proliferation and Migration Assays.** Real-time cell proliferation assays were performed on xCELLigence system from ACEA Biosciences. Briefly, for proliferation experiments, 100 μL of cell culture media was added to each well of E-Plate to obtain background readings. 5×10^3^ cells in 100 media were then seeded into the E-Plate. The E-Plates containing cells were then incubated for 30 minutes at room temperature and placed on the RTCA DP instrument located in a cell culture incubator. The Cell Index (CI) values were measured automatically every 15 minutes for 5 days. For the migration experiments, the lower chamber of CIM plate was filled with serum-containing media and the upper chamber was filled with serum-free media. Cells were resuspended in serum-free medium, counted and seeded in the upper chamber applying 5-10×10^4^ cells in 100 µL. After cell addition, CIM plate was incubated 30 minutes at room temperature and then blocked in the RTCA DP instrument. The CI values were measured automatically every 15 minutes for 48 h.

**Wound Healing Assay.** Cells were plated in 6-well plates and allowed to grow to 90% confluence. The medium was then removed, and wounds with 10μl pipette tip. Floating cells were removed before complete medium was added. The wound healing process was monitored under a microscope. Each assay was repeated 3 times.

**Lentiviral Packaging and Cell Transduction.** Short hairpin RNA (shRNA) targeting PEAK1 and miR-181d precursor sequences were cloned into pLenti-U6-GFP and pLenti-Puro-MIR (ViGeneBio, Shandong, China) respectively. Wild type KRas were PCR amplified and sub-cloned into GV367 lentiviral vectors (Genchem, Shanghai, China). Lentivirus were amplified by transfecting HEK-293T cells with PMD2G and PSPAX2 packaging plasmids and the corresponding backbone plasmid using Lipofectamine 2000 (Invitrogen) according to the manufacturer’s instruction. Eight hours after transfection, the medium was refreshed. 48 hours later, the supernatant was harvested, and CRC cells were infected in the presence of 5 µg/mL polybrene for 12 hours, and the medium was refreshed. 72 hours post the infection, the efficiency of infection was measured under a fluorescent microscope.

**Luciferase Assays.** The 3’UTR segment of the PEAK1 gene was ampliﬁed by polymerase chain reaction (PCR). The PCR primers for the PEAK1 3’UTR containing the miR-181d target site (Forward: 5’-cccggg gagctc TTCCCAGAGACAGGTGGCTA-3’; Reverse: 5’- gggccc acgcgt CAAGACTAGGTTACCACTCTA-3’). A mutant construct in miR-181d recognition site (ATCC to CCGG mutation) of PEAK1 3’UTR region also was generated using specific primer (Forward: 5’-cccggg gagctc TTCCCAGAGACAGGTGGCTA-3’; Reverse: 5’- gggccc acgcgt TCTGCTATTGTAAGAGTCTTGCTC-3’). The 3’UTR cDNA was inserted into the pMIR-reporter (Promega, Madison, WI, USA). Co-transfections of PEAK1 3’UTR or mut PEAK1 3’UTR plasmid with miR-181d mimics into the HCT 116 cells were accomplished by using Lipofectamine 2000 (Invitrogen). Luciferase activity was measured 48 hours after transfection by the Dual-Luciferase Reporter Assay System (Promega). Each assay was repeated in 4 independent experiments.

**Microarray Expression Profiling.** HCT 116 cells were tranfected with siRNA or NC for 48 hours. The total RNA of transfected cells was extracted by TRIzol. Sample labeling and array hybridization were performed according to the Agilent One-Color Microarray-Based Gene Expression Analysis protocol (Agilent Technology).

**Statistical Analysis****.** Statistical analysis was done using the SPSS software 18.0 (SPSS, IBM, Chicago, IL, USA). Data are expressed as the means ± SD. Statistical significance was determined using Student’s t-test or Mann-Whitney U test as appropriate. The correlations between PEAK1/miR-181d expression levels and the clinicopathologic parameters of the CRC patients were evaluated by Chi-square Test. Survival curves were generated using the Kaplan-Meier method and assessed using the log-rank test. The independent prognostic factors were identified by performing Multivariate Cox regression analysis. Associations between the expression levels of the two targets were analyzed using the Spearman’s rank correlation coefficient.

# Supplementary References

1. Wen C, Chen J, Zhang D, Wang H, Che J, Qin Q*, et al.* Pseudolaric acid B induces mitotic arrest and apoptosis in both 5-fluorouracil-sensitive and -resistant colorectal cancer cells. *Cancer Lett* 2016, **383**(2)**:** 295-308.

2. Song M, Chen D, Lu B, Wang C, Zhang J, Huang L*, et al.* PTEN loss increases PD-L1 protein expression and affects the correlation between PD-L1 expression and clinical parameters in colorectal cancer. *PloS one* 2013, **8**(6)**:** e65821.

3. Xavier LL, Viola GG, Ferraz AC, Da Cunha C, Deonizio JM, Netto CA*, et al.* A simple and fast densitometric method for the analysis of tyrosine hydroxylase immunoreactivity in the substantia nigra pars compacta and in the ventral tegmental area. *Brain Res Brain Res Protoc* 2005, **16**(1-3)**:** 58-64.

# Supplementary Figure

# S1

**
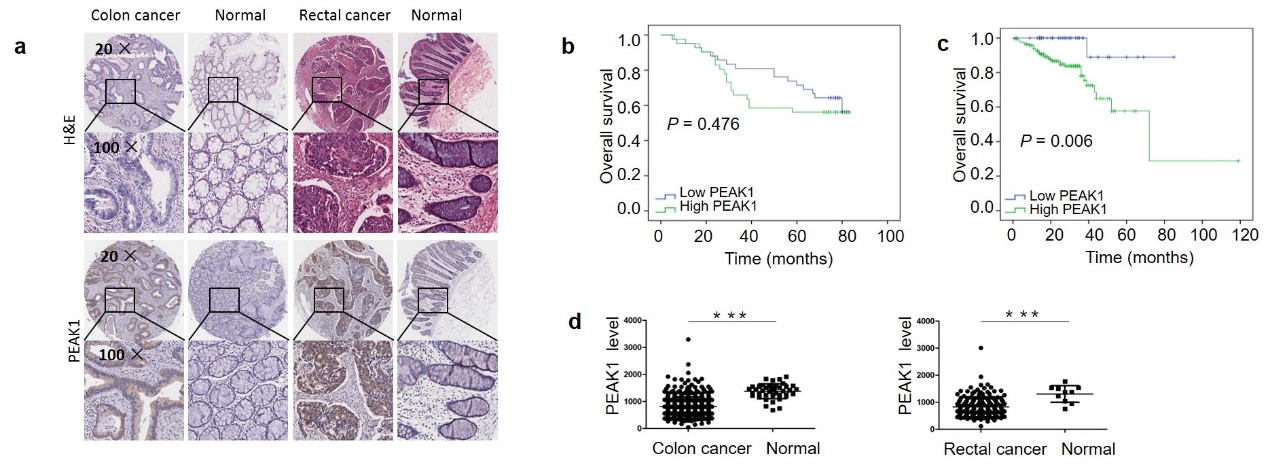
**

**Figure S1.** PEAK1 expression levels in CRC. (**a**) Representative immunohistochemical images were taken at different magnifications in cancer tissues and tumor-adjacent normal tissues. (**b**) Kaplan–Meier analysis of overall survival according to low and high PEAK1 protein expression in 83 rectal cancer patients. (**c**) The PEAK1 expression data were obtained from TCGA RNAseqV2. Kaplan–Meier analysis of overall survival according to low and high PEAK1 protein expression in 264 CRC patients. (**d**) The PEAK1 expression data were obtained from TCGA RNAseqV2. PEAK1 expression was dramatically reduced in colon and rectal cancer tissues. Statistical significance was determined by a two-tailed unpaired Student’s *t* test.

# S2


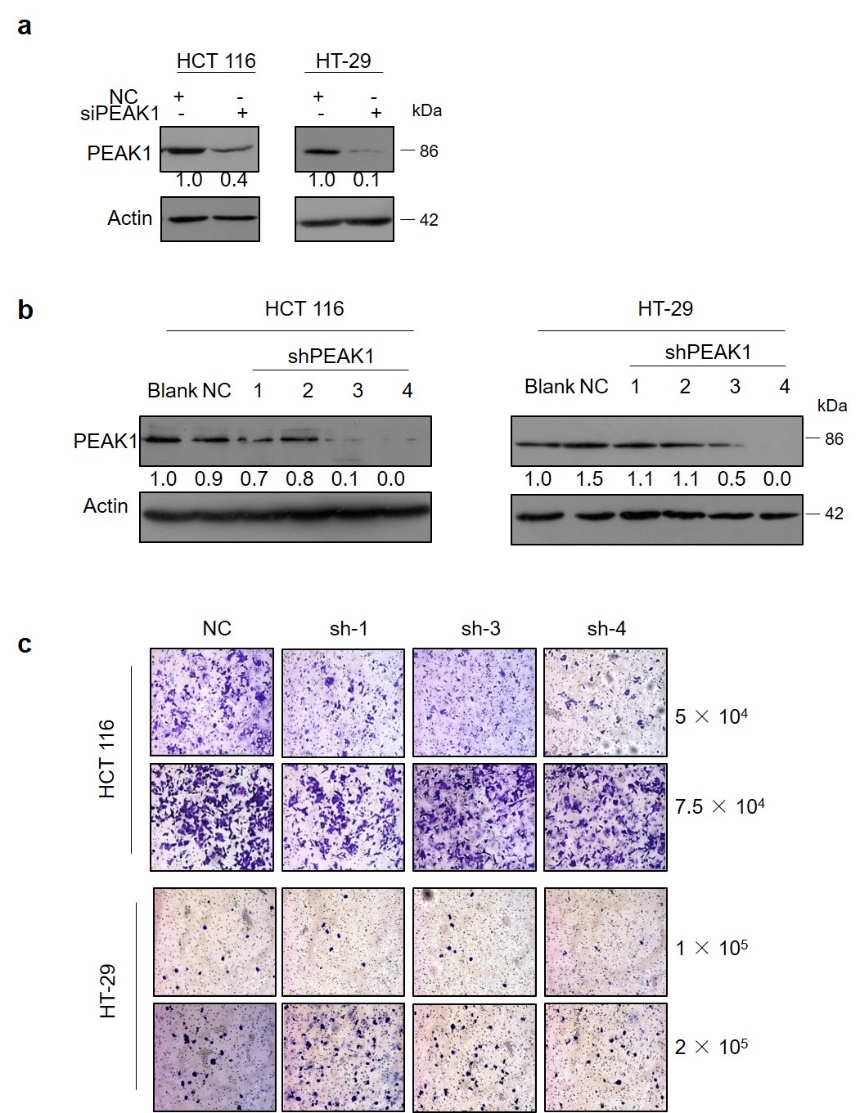


**Figure S2.** Down-regulation of PEAK1 inhibits CRC cell invasion. (**a**) Transfection of HCT 116 and HT-29 cells with siPEAK1 causes decreased PEAK1 protein expression. (**b**) HCT 116 and HT-29 cells infected with pLenti-shPEAK1 exhibited decreased PEAK1 protein expression. (**c**) Transwell assays were used to estimate the effects of PEAK1 down-regulation on CRC cell invasion abilities.

# S3


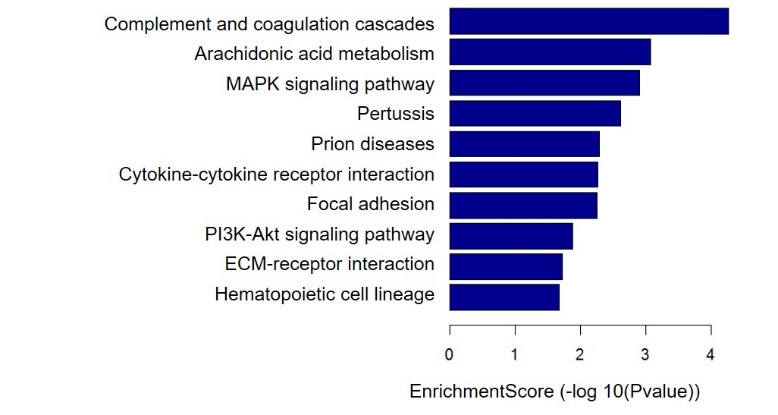


**Figure S3.** PEAK1 is involved in the EGFR signaling pathway. The top 10 pathways that were down-regulated in siPEAK1 compared to the NC.

# S4


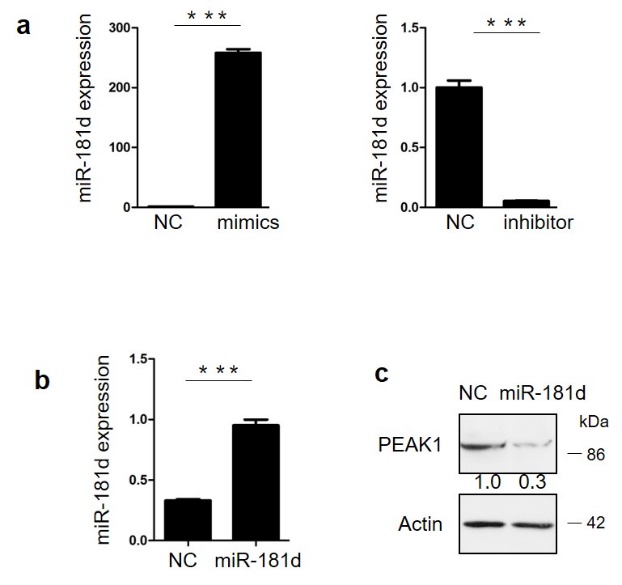


**Figure S4.** Overexpression of miR-181d inhibits the expression of PEAK1. (**a**) qRT-PCR analysis of the expression of miR-181d in HCT 116 cells transfected with miR-181d mimics or inhibitors. (**b**) HCT 116 cells infected with plenti-miR-181d or pLenti-vector. (**c**) Western blot showed inhibition of PEAK1 levels by miR-181d. (Data are represented as the mean ± SD. ***P < 0.001).

# Supplementary Tables

**Table S1** Univariate and multivariate analyses of various potential prognostic factors in 83 rectal cancer patients.

| **Factors** | **Univariate analysis** | | **Multivariate analysis** | |
| --- | --- | --- | --- | --- |
|  | **HR (95%CI)** | ***P*** | **HR (95%CI)** | ***P*** |
| **Age** | 1.037 (0.998, 1.078) | 0.063 | 1.047 (1.009, 1.087) | 0.016 |
| **Gender** | 1.144 (0.578, 2.266) | 0.700 |  |  |
| **TNM Stage** | 1.882 (1.206, 2.937) | 0.005 | 1.882 (1.206, 2.937) | 0.005 |
| **PEAK1 expression** | 4.407 (0.823, 23.607) | 0.083 |  |  |

HR, hazard ratio

CI, confidence interval

**Table S2** Correlation between miR-181d expression and clinical parameters in 353 CRC patients.

| **Factors** | **n** | **miR-181d level** | | ***P* value** |
| --- | --- | --- | --- | --- |
|  |  | **Low (%)** | **High (%)** |  |
| **Age** |  |  |  | 0.138 |
| <60 | 164 | 95 (57.9) | 69 (42.1) |  |
| ≥60 | 189 | 124 (65.6) | 65 (34.4) |  |
| **Gender** |  |  |  | 0.331 |
| Male | 196 | 126 (64.3) | 70 (35.7) |  |
| Female | 157 | 93 (59.2) | 64 (40.3) |  |
| **Tumor size** |  |  |  | 0.331 |
| <5cm | 175 | 113 (64.6) | 62 (35.4) |  |
| ≥5cm | 178 | 106 (59.6) | 72 (40.4) |  |
| **Tumor location** |  |  |  | 0.032* |
| Colon | 174 | 98 (56.3) | 76 (43.7) |  |
| Rectum | 178 | 120 (67.4) | 58 (32.6) |  |
| **Local relapse** |  |  |  | 0.044* |
| Yes | 15 | 13 (86.7) | 2 (13.3) |  |
| No | 337 | 205 (60.8) | 132 (39.2) |  |
| **TNM stage** |  |  |  | 0.040* |
| Ⅰ | 58 | 42 (72.4) | 16 (27.6) |  |
| Ⅱ | 140 | 81 (57.9) | 59 (42.1) |  |
| Ⅲ | 123 | 71 (57.7) | 52 (42.3) |  |
| Ⅳ | 29 | 23 (79.3) | 6 (20.7) |  |

**P* < 0.05, Chi-square test

**Table S3** Univariate and multivariate analyses of various potential prognostic factors in 353 CRC patients.

| **Factors** | **Univariate analysis** | | **Multivariate analysis** | |
| --- | --- | --- | --- | --- |
|  | HR (95%CI) | *P* | HR (95%CI) | *P* |
| **Age** | 0.75 (0.50, 1.12) | 0.159 |  |  |
| **Gender** | 1.13 (0.76, 1.67) | 0.557 |  |  |
| **Tumor size** | 1.18 (0.79, 1.75) | 0.421 |  |  |
| **Tumor location** | 1.36 (0.91, 2.02) | 0.133 |  |  |
| **Local relapse** | 1.73 (0.80,3.74) | 0.164 |  |  |
| **TNM stage** | 3.21 (2.12, 4.89) | <0.001* | 3.19 (2.06, 4.93) | <0.001* |
| **Grade** | 0.53 (0.35, 0.79) | 0.002* | 0.52 (0.31, 0.88) | 0.015* |
| **miR-181d expression** | 0.65 (0.42, 0.99) | 0.047* | 0.60 (0.39, 0.94) | 0.024* |

**P* < 0.05; HR, hazard ratio; CI, confidence interval.

**Table S4** Correlation between PEAK1 expression and clinical parameters in 264 CRC patients.

| **Factors** | **n** | **Low PEAK1**  **expression (%)** | **High PEAK1**  **expression (%)** | ***P* value** |
| --- | --- | --- | --- | --- |
| **Age** |  |  |  | 0.060 |
| <60 | 52 | 16 (30.8) | 36 (69.2) |  |
| ≥60 | 212 | 40 (18.9) | 172 (81.1) |  |
| **Gender** |  |  |  | 0.763 |
| Male | 132 | 29 (22.0) | 103 (78.0) |  |
| Female | 132 | 27 (20.5) | 105 (79.5) |  |
| **Tumor location** |  |  |  | 0.523 |
| Colon | 192 | 39 (20.3) | 153 (79.7) |  |
| Rectum | 71 | 17 (23.9) | 54 (76.1) |  |
| **TNM stage** |  |  |  | 0.549 |
| I | 53 | 12 (22.6) | 41 (77.4) |  |
| II | 100 | 17 (17.0) | 83 (83.0) |  |
| III | 69 | 18 (26.1) | 51 (73.9) |  |
| IV | 41 | 9 (22.0) | 32 (78.0) |  |
| **Vascular invasion** |  |  |  | 0.609 |
| Yes | 61 | 13 (21.3) | 48 (78.7) |  |
| No | 164 | 30 (18.3) | 134 (81.7) |  |
| **Lymphovascular invasion** |  |  |  | 0.150 |
| Yes | 135 | 31 (23.0) | 104 (77.0) |  |
| No | 109 | 17 (15.6) | 92 (84.4) |  |
| **Relapse** |  |  |  | 0.187 |
| Yes | 33 | 5 (15.2) | 28 (84.8) |  |
| No | 146 | 38 (26.0) | 108 (74.0) |  |

**Table S5** Univariate and multivariate analyses of various potential prognostic factors in 264 CRC patients.

| **Factors** | **Univariate analysis** | | **Multivariate analysis** | |
| --- | --- | --- | --- | --- |
|  | **HR (95%CI)** | ***P*** | **HR (95%CI)** | ***p*** |
| Age | 1.036 (1.000, 1.074) | 0.051 |  |  |
| Gender | 0.641 (0.321, 1.282) | 0.209 |  |  |
| Tumor location | 1.130 (0.508, 2.510) | 0.765 |  |  |
| MS | 1.866 (0.838, 4.158) | 0.127 |  |  |
| Vascular | 1.601 (0.738, 3.473) | 0.233 |  |  |
| Lymphovascular | 2.209 (1.028, 4.745) | 0.042 |  |  |
| TNM Stage | 2.751 (1.838, 4.120) | <0.001 |  |  |
| Relapse | 10.081 (3.870, 26.259) | <0.001 | 12.606 (4.532, 35.066) | <0.001 |
| PEAK1 expression | 9.716 (1.327, 71.163) | 0.025 |  |  |

HR, hazard ratio

CI, confidence interval

# Grey value of Western blot

**Figure 3b**

**
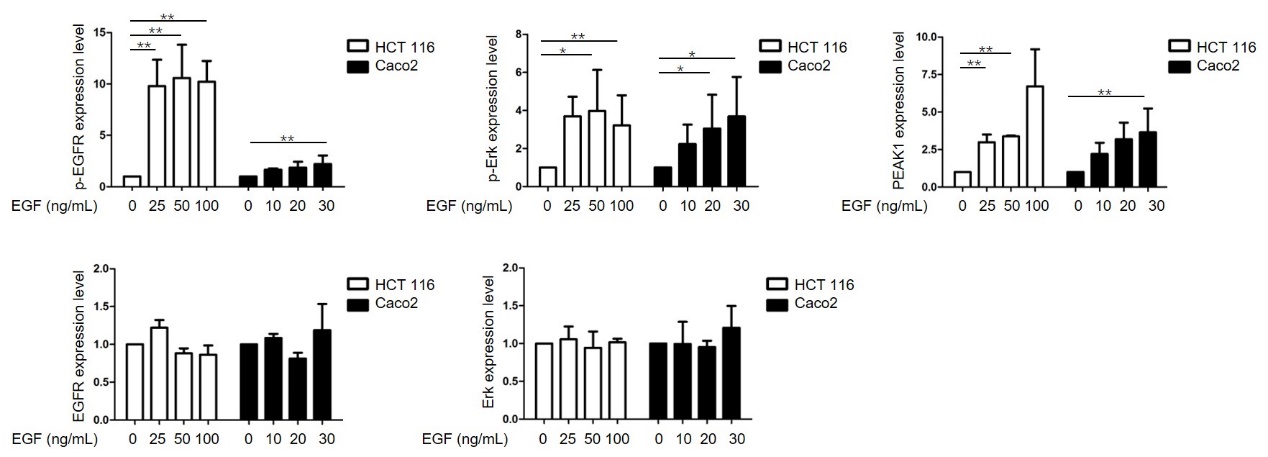
**

**Figure 3c**

**
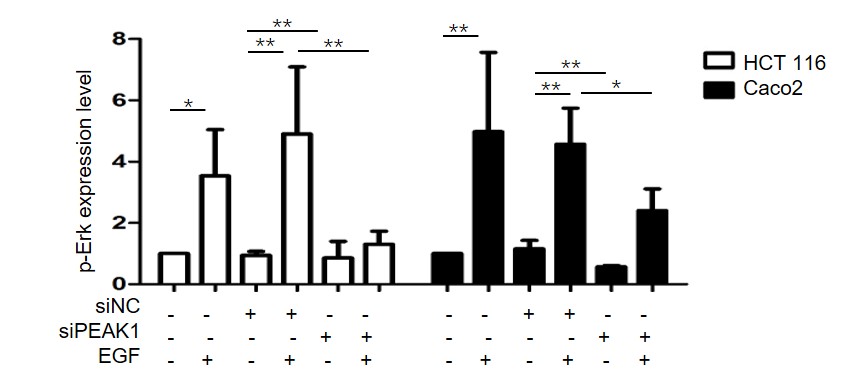
**

**Figure 4c**

**
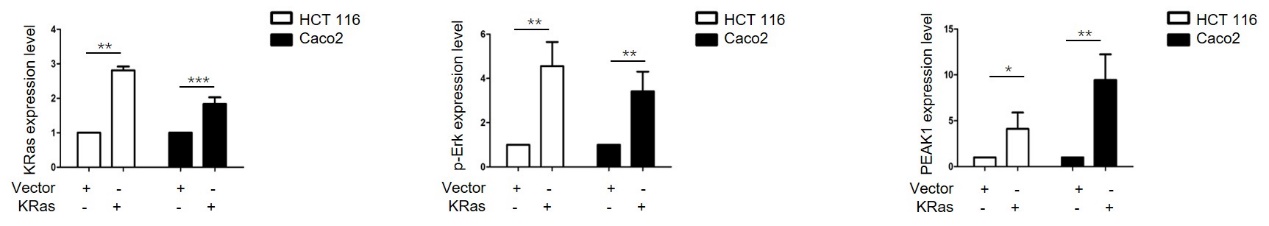
**

**Figure 4e**

**
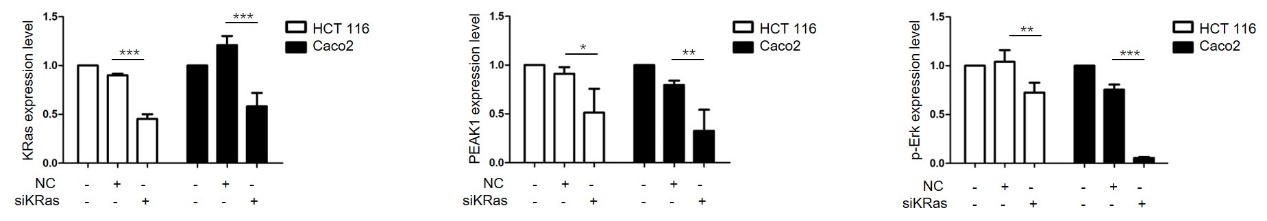
**

**Figure 6c**

**
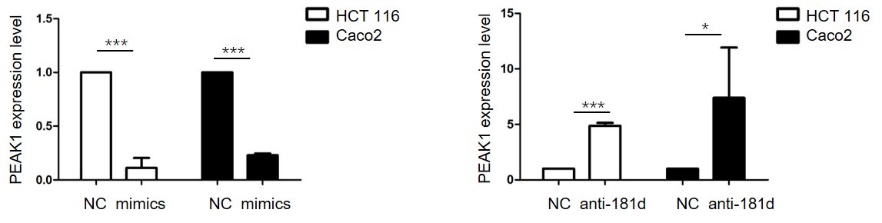
**

**Figure 6d**

**
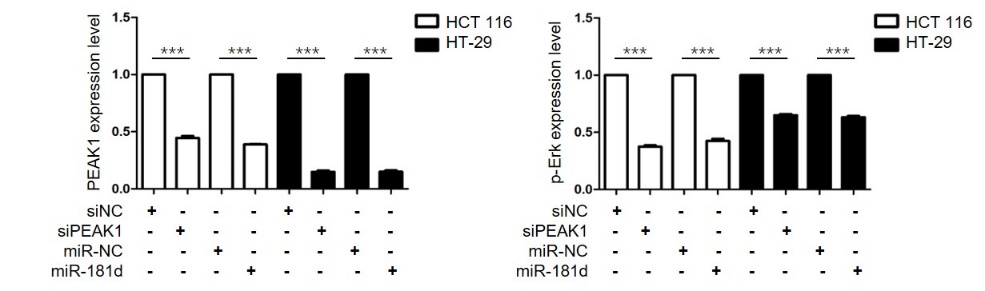
**
